# Immune Restoration Disease in HIV Patient

**DOI:** 10.3201/eid1204.050455

**Published:** 2006-04

**Authors:** Neil E. Jenkins, Mike B.J. Beadsworth, James J. Anson, Fred J. Nye, Vanessa J. Martlew, Nick J. Beeching

**Affiliations:** * Liverpool School of Tropical Medicine, Liverpool, United Kingdom

**Keywords:** Mycobacterium microti, HIV, immunosuppression, AIDS-related opportunistic infections, immune restoration disease, Dispatch

## Abstract

We describe a severely immunosuppressed HIV-1–positive man in whom immune restoration disease associated with pulmonary infection caused by *Mycobacterium microti* developed after antiretroviral treatment. The diagnosis was made by using convenient spoligotyping techniques, but invasive investigations were required to exclude a tumor.

During the first few months of highly active antiretroviral treatment, immune restoration may be complicated by clinical events in which either previously subclinical infections are found or preexisting partially treated opportunistic infections deteriorate. This condition, termed immune restoration disease (IRD), is thought to be caused by the improvement in the host's immune response to pathogens. We report what we believe is the first recorded case of IRD with confirmed *Mycobacterium microti* infection. *M. microti* is an unusual infection associated with small rodents. Novel genetic techniques have confirmed it as a human pathogen, but its true incidence remains unclear.

## Case Report

A 33-year-old man was admitted to the hospital in 2002 with a 3-month history of intermittent hemoptysis. He had previously visited hematology outpatient clinics for many years for hemophilia A (3%–5% factor VIII) and HIV-1 had been diagnosed in 1996 (a stored sample from 1989 retrospectively tested HIV-1–antibody positive). CD4 cell counts had been recorded as 434 × 10^6^/L in 1996 and 260 × 10^6^/L in 1998. After this time, he failed to attend any further clinic appointments.

**Figure 1:**
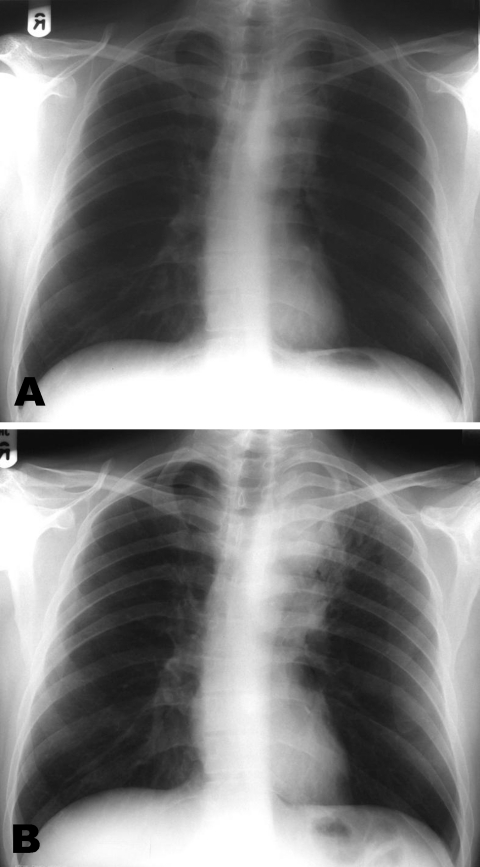
Chest radiographs at initiation of A) highly active antiretroviral therapy (HAART) showing left hilar mass; and B) after 9 weeks of HAART and antituberculosis treatment, suggesting enlargement of the hilar mass consistent with immune restoration disease. (The radiograph has been flipped horizontally to aid comparison).

In addition to worsening hemoptysis, he had minor weight loss but no night sweats. He appeared moderately unhealthy, with oral thrush and mild splenomegaly. A chest radiograph showed left upper lobe shadowing (Figure 1, panel A), sputum smears contained numerous acid-fast bacilli, and sputum was sent for culture, speciation, and sensitivity testing. Blood tests showed a CD4 cell count of 6 × 10^6^/L and HIV viral load of 272,000 (log 5.4) copies/mL. His hemoglobin was 12.1 g/dL, leukocyte count 4.9 × 10^9^/L, and platelets 367 × 10^9^/L. Renal and liver function test results were normal, apart from a γ-glutamyltransferase level of 200 U/L (normal <35 U/L). An ultrasound scan of his abdomen showed a diffuse increase in liver echogenicity and an enlarged (13.6-cm) spleen. He was naturally immune to hepatitis B, and tests for hepatitis C antibody and viremia were negative.

He was given broad-spectrum antimycobacterial therapy (rifampin, isoniazid, pyrazinamide, ethambutol, and azithromycin) as treatment for both *M. tuberculosis* complex (MTB complex) and opportunistic mycobacteria. He improved throughout a 2-week hospital stay and highly active antiretroviral therapy (HAART) was begun. It consisted of zidovudine and lamivudine and an increased dose of efavirenz (800 mg daily) because of concomitant rifampicin therapy, together with cotrimoxazole for prophylaxis against *Pneumocystis jirovecii*.

**Figure 2:**
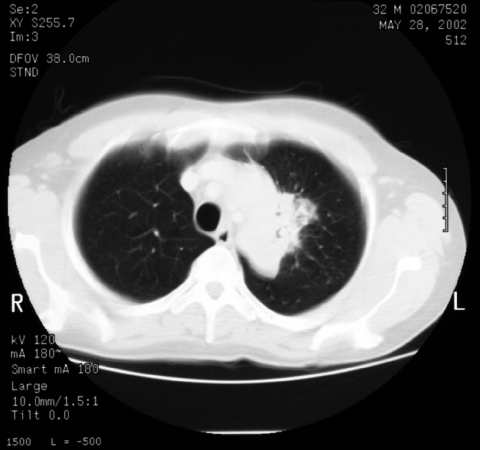
Computed tomographic scan of thorax at patient's initial visit. Results are highly suggestive of tumor.

At a 3-week follow-up appointment, the patient had continued to improve. No further hemoptysis had occurred, and he had gained 2 kg body weight. After that, by telephone, he complained of occasional fevers and malaise but declined to be seen. Nine weeks after his initial visit, he came to the ward and with symptoms of breathlessness, nausea, and diarrhea. He had fevers spiking to >38°C, and newly palpable lymph nodes were evident in the right axilla and over the left parotid gland. A chest radiograph showed increased shadowing (Figure 1, panel B) and a computed tomographic (CT) scan of the chest showed a 6 × 3-cm solid mass in the upper left chest with visible air bronchograms and minor volume loss, reported as "almost certainly solid neoplasm" (Figure 2). A bronchoscopy, before which the patient received factor VIII, showed an area of irregular nodular tissue in the posterior segment of the left upper lobe. His CD4 count was 26 × 10^6^/L.

While we awaited histologic examination results, the patient's medication was withdrawn, and he improved within days. The lymph nodes reduced in size, and the spiking temperature resolved. Eight weeks after admission, the results of IS*6110* polymerase chain reaction (PCR) testing on the slow-growing, positive mycobacterial culture confirmed the presence of MTB complex. The bronchial biopsy specimen showed necrotizing granulomatous bronchitis; acid-fast bacilli were not visible, and this specimen did not grow any mycobacteria.

The *Mycobacterium* species was not growing well in the laboratory, but *M. microti* was identified in the culture with spoligotyping methods (*1**,**2*). On extension of culture, the isolate was confirmed as *M. microti* phenotypically and found to be sensitive in vitro to rifampin, isoniazid, ethambutol, and clarithromycin but resistant to pyrazinamide.

The antimycobacterial drugs were reintroduced without problems 4 weeks later, as was HAART after an additional 8 weeks. Thirty months after restarting antimycobacterial therapy, the patient remained well with a total weight gain of 19 kg, complete radiologic resolution of the pulmonary mass, undetectable HIV viral load (<50 copies/mL), and a progressive rise in his CD4 count to 249 × 10^6^/L.

## Conclusions

*M. microti* is a slow-growing member of the MTB complex. It has most commonly been described in association with small rodents (*2**,**3*). Its role as a pathogen in humans has been proposed by a handful of case reports involving both immunocompetent and immunodeficient persons (*4**–**6*). Because of the slow growth of the bacterium, confirmatory diagnosis is difficult, and cultures may be wrongly discarded as negative after a routine 8- to 12-week culture period. In such cases several commercial tests can identify the organism to the level of the MTB complex. The members of the tuberculosis complex are not reliably distinguished on biochemical grounds. Restriction fragment length polymorphism, DNA fingerprinting, is a time-consuming, slow, and expensive method of distinguishing members. Spoligotyping (spacer oligotyping) is a PCR-based test that can be quickly performed, even on the small amounts of DNA in poorly growing cultures. Strains differ in the size of spacer regions that intersperse direct repeat regions. These polymorphisms create differences in the PCR products produced, a process that allows strain identification (*1**,**7*). Other PCR-based tests are being developed that may have advantages over spoligotyping for identifying some members of the complex (*8*). Culture remains the standard and allows drug sensitivity testing. We suggest that such cultures be extended for up to 6 months if acid-fast bacilli are noted in specimens from a patient with consistent pulmonary pathologic findings.

Severe immunosuppression associated with HIV-1 can allow coinfections to remain subclinical. After HAART is introduced, IRD can lead to the development of opportunistic infections with atypical and severe clinical signs and symptoms. In this case, necrotizing granulomatous inflammation occurred without isolation of mycobacteria, which is atypical in an immunodeficient HIV patient. These clinical manifestations have been described in many diseases, including MTB and other opportunistic mycobacterial infections (*9*), herpesvirus infections, and hepatitis B and C (*10*). Similar granulomatous endobrochial lesions have been described during immune restoration with *M. avium* complex in HIV-infected patients (*11*). IRD has been described as occurring in 30% to 40% of those with very low CD4 counts when they begin HAART, developing within the first few months of treatment as the CD4 count rises. IRD is a diagnosis of exclusion: other processes such as drug fever, resistance, treatment failure, or other infections must be excluded. IRD may be a life-threatening condition, and clinical interventions, such as stopping medications, steroids, or both, may be indicated. An associated increase in markers of immune activation occurs, which seems to vary depending on the pathogen involved (*12**,**13*).

In MTB, the major risk factor for IRD is beginning HAART within 2 months of antituberculosis treatment. This fact has led many experts to recommend delaying HAART for 2 months, a strategy that may also reduce the incidence of adverse events. However, a recent retrospective review suggested that delaying HAART would, particularly in those with CD4 cell counts<100 cells × 10^6^/L, lead to significant increase in risk for death and new opportunistic infections (*14*). IRD should always be considered along with treatment failure if a patient's clinical condition deteriorates after HAART is introduced.
